# Wearable SIMO Inductive Resonant Link for Posture Monitoring

**DOI:** 10.3390/s25175478

**Published:** 2025-09-03

**Authors:** Giuseppina Monti, Daniele Lezzi, Luciano Tarricone

**Affiliations:** Department of Engineering for Innovation, University of Salento, 73100 Lecce, Italy; daniele.lezzi@studenti.unisalento.it (D.L.); luciano.tarricone@unisalento.it (L.T.)

**Keywords:** inductive resonant coupling, posture monitoring, wearable, textile materials, single input multiple output

## Abstract

This paper explores the feasibility of using a wireless Inductive Resonant Link (IRL) for wearable posture monitoring. The proposed system is based on magnetically coupled textile resonators and is implemented using a Single Input Multiple Output (SIMO) configuration. In particular, the setup consists of four inductively coupled resonators: one transmitting coil integrated into a textile structure and positioned on the back of the neck, and three receiving coils placed on the shoulders. The magnetic coupling between these elements varies as a function of the user’s posture, making it possible to monitor postural changes by analyzing variations in the transmission coefficients of the link. Unlike traditional sensor-based systems that require multiple components and data processing, the proposed method uses the inherent response of the inductive link to detect posture in a simple and efficient way. To validate the concept, experimental measurements of the scattering parameters were carried out using a compact and low-power vector network analyzer. The results show a consistent and measurable relationship between postural changes and variations in the transmission coefficients, demonstrating the effectiveness of the proposed system in distinguishing between different postures. The findings suggest that inductive resonant wireless links, especially when implemented with textile components, represent a promising alternative to traditional wearable sensor technologies for posture tracking. The approach offers significant advantages in terms of wearability, power consumption, and simplicity, making it suitable for applications in ergonomics, rehabilitation, occupational health, and smart clothing.

## 1. Introduction

Nowadays, human movement recognition plays a critical and increasingly prominent role in many fields. For instance, in sports science, it is fundamental for training and evaluating the physical performance of athletes, where monitoring posture and body composition directly impacts motor abilities and injury prevention [1,2].

Similarly, finger and hand gesture recognition systems are pivotal for enhancing human–computer interaction, especially in assistive devices and intuitive control systems [3,4].

Moreover, real-time recognition tasks using wearable sensors have been successfully applied to human–machine interfaces in virtual reality and gaming environments, allowing for natural and immersive experiences [5,6].

Among these applications, the monitoring of neck posture has recently gained particular importance. Maintaining a correct neck posture is essential to prevent musculoskeletal disorders (MSDs), which can significantly impair an individual’s quality of life and work productivity [7,8,9,10]. The neck is especially vulnerable to strain caused by prolonged poor posture, often related to the widespread use of smartphones and other handheld devices. Musculoskeletal disorders encompass a variety of inflammatory and degenerative conditions characterized by symptoms such as pain, stiffness, tingling, and numbness in affected areas [11,12,13]. Specifically, the connection between smartphone usage and neck disorders has been the subject of recent research. For example, a study conducted on 313 university students found that 46% of those identified as “smartphone-addicted” experienced neck disorders, underlining the critical role of device use habits in musculoskeletal health [11]. This is supported by further epidemiological data indicating a high prevalence of text neck posture and related musculoskeletal complaints among young adults, which have been exacerbated by increased screen time during the COVID-19 pandemic [11,12]. These findings highlight the urgent need for effective, non-invasive posture monitoring systems that can provide real-time feedback and help mitigate the risks associated with poor neck positioning.

In this context, wearable and sensor-based technologies represent a promising solution for continuous, unobtrusive monitoring of neck posture [14]. The ability to accurately detect posture changes in real time can empower users to correct harmful habits and potentially reduce the incidence of MSDs.

Accordingly, many techniques have been proposed in the literature for human movement recognition, which can broadly be classified into two main categories: vision-based and sensor-based techniques. Each of these approaches comes with its own advantages and limitations, making them suitable for different application scenarios.

Vision-based techniques rely on one or more cameras to capture video or image sequences of the user’s movements. The captured visual data are then processed through advanced image analysis and computer vision algorithms to extract meaningful information about human motion [6]. These methods can provide rich spatial and temporal details about movement, enabling highly accurate recognition of complex gestures and postures. However, vision-based systems are highly dependent on environmental conditions. For instance, their performance can be significantly affected by ambient lighting variations, shadows, or occlusions. Additionally, these systems require a clear line-of-sight between the cameras and the subject, which limits their applicability in cluttered or dynamic environments. Due to these constraints, vision-based approaches are generally unsuitable for developing portable, wearable solutions that can function reliably in any environment.

Sensor-based methods, on the other hand, have gained substantial popularity in recent years, especially with the rapid advancement of wearable technology and miniaturized electronics. These methods involve integrating electronic sensors—such as accelerometers, gyroscopes, magnetometers, inertial measurement units (IMUs), light emitting diodes (LEDs), and textile-based sensors—into wearable garments or accessories [14,15,16,17]. Sensor-based systems offer several advantages, including portability, continuous monitoring capabilities, and the possibility of real-time data acquisition without requiring a fixed setup. Nevertheless, these systems are not without challenges. Data collected from sensors often require extensive processing, such as integration and filtering, which can introduce errors or drift over time. Moreover, certain sensors are sensitive to environmental disturbances: for example, magnetometers are vulnerable to ferromagnetic interference from nearby metallic objects; gyroscopes can be affected by mechanical vibrations and shocks; IMUs may lose accuracy during rapid or complex movements [18,19]. Another important limitation is related to the necessity of multiple electronic components for signal acquisition and processing, which increases power consumption, complexity, and may hinder seamless integration into everyday wearable devices.

Within the realm of sensor-based solutions, textile sensors have emerged as a particularly promising option for enhancing wearability and comfort in motion monitoring devices. Textile sensors can be seamlessly embedded into clothing, offering unobtrusive and flexible sensing capabilities [14,18,20]. These sensors are generally categorized into three types: resistive, capacitive, and inductive.

Resistive textile sensors operate by detecting changes in electrical resistance caused by mechanical deformation, such as stretching or bending. Despite their simplicity and ease of fabrication using various conductive materials and patterns, resistive sensors suffer from drawbacks like high hysteresis, non-linear responses, and signal drift under prolonged use or repeated stretching, which can compromise measurement accuracy.

Capacitive textile sensors consist of a dielectric layer sandwiched between conductive electrodes, measuring changes in capacitance as the sensor deforms elastically in response to external forces. These sensors typically provide improved performance over resistive types in terms of hysteresis and linearity. However, their reliability can be affected by mechanical mismatches between the dielectric and conductive layers, potentially causing interfacial cracks, layer separation, or delamination over time [16,17,21,22,23,24].

Inductive textile sensors detect changes in inductance caused by three-dimensional deformation of planar inductors, which can be fabricated through embroidery techniques using conductive threads on elastic textile substrates [14,20,25,26,27]. These sensors combine the advantages of capacitive sensors with enhanced mechanical robustness, as the use of homogeneous embroidery materials minimizes issues related to material mismatches. Inductive sensors, thus, offer a promising balance between sensitivity, durability, and ease of integration into garments.

Another wearable sensing solution gaining attention is chipless RFID (Radio Frequency Identification) technology [28,29,30,31,32,33]. Chipless RFID tags eliminate the need for integrated electronics by encoding information through passive electromagnetic structures printed or sewn onto fabrics. These tags interact with a reader antenna that transmits RF signals and processes the backscattered response to detect movements or gestures. While chipless RFID offers excellent wearability and manufacturing flexibility, it faces challenges such as potential interference between multiple tags, noise in harsh environments, and the dependence on an external reader antenna. This limits its use to suitably equipped environments and restricts user mobility.

A relevant implementation of passive magneto-inductive sensing is described in [34], where passive tag coils—constructed using high-frequency litz wire wound onto 3D-printed rigid frames—are placed on limbs and the head, while four active anchor coils on the torso are connected to PCBs with impedance matching networks, switches, and external processing hardware. The system measures impedance variations across these coils and requires offline data processing. However, the system requires multiple fixed anchor coils and a central processing unit, which increases the overall complexity and limits wearability. Moreover, the use of rigid 3D-printed structures and wire-wound solenoids further reduces comfort and makes the solution less suitable for integration into garments or extended daily use.

Building on these concepts, an alternative approach that stands out involves the use of textile inductors within wireless inductive resonant links (IRLs) [35]. IRLs exploit the magnetic coupling between resonators for wireless transmission and have been extensively studied in Wireless Power Transfer (WPT) applications [36,37,38]. They offer significant advantages, including high efficiency over low- to mid-range distances and robustness against environmental factors [39]. However, IRLs exhibit a key limitation: their performance strongly depends on the alignment between transmitting and receiving resonators. Misalignments reduce magnetic coupling and thereby degrade the transmission coefficient [40].

Interestingly, this dependency on alignment can be turned into an advantage. By strategically positioning resonators around a joint, the variation in magnetic coupling can be harnessed for precise movement recognition. This makes IRLs particularly suitable for posture monitoring applications, as they do not require line-of-sight, minimizing the need for complex electronics and simplifying data processing.

Accordingly, a system based on a Single Input Multiple Output (SIMO) wireless inductive resonant link for wearable posture monitoring is proposed. The specific objective is to demonstrate the feasibility of this approach through a preliminary prototype. Direct measurements of scattering parameters were performed for different neck postures relative to the shoulders, showing that the measured values can be clearly associated with specific postural configurations. This proof-of-concept aims to establish the foundation for further developments in wearable posture sensing using inductive coupling technologies.

The proposed system employs textile-based inductors realized by using a conductive non-woven fabric combined with a soft textile substrate, ensuring high comfort and mechanical adaptability suitable for continuous wear. The electronic components are compact and modular, connected to the textile sensors via snap buttons, allowing easy removal for washing or replacement. In future wearable implementations, a miniaturized measurement board—essentially a simplified version of a vector network analyzer without a display—could be embedded within the garment. This compact electronics module would acquire scattering parameters and either process them locally with an embedded microcontroller or wirelessly transmit data to an external device, enabling real-time posture monitoring while maintaining high usability and wearability.

The paper is structured as follows. Section 1 and Section 2 present the operating principle of the proposed system and the implementation analyzed in this paper. Section 3 presents and discusses the achieved experimental results. Finally, some conclusions are drawn in Section 4.

## 2. Proposed System and Implementation for Posture Monitoring

### 2.1. Inductive Resonant Wireless Link

Inductive Resonant (IR) wireless links rely on magnetic coupling between resonators and represent a well-established solution for short-range wireless energy transmission. The simplest configuration consists of two magnetically coupled resonators, which can be modeled using classical network theory as a two-port network.

**Figure 1:**
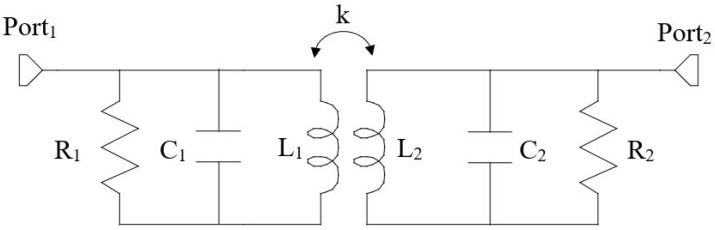
Equivalent circuit of an inductive resonant wireless link consisting of two magnetically coupled resonators in shunt configuration.

Each resonator comprises a distributed inductor and a lumped capacitor, connected either in series or parallel. An equivalent circuit for the parallel configuration is shown in Figure 1. To ensure maximum energy transfer, both resonators must be tuned to resonate at the same frequency $f_{0}$, determined by the inductance and capacitance values as follows:

(1) $$f_{0}=\frac{1}{2\pi \sqrt{L_{i}C_{i}}},\;i=\left\{1,2\right\}$$

The resistive losses within the resonators, primarily attributed to conductor losses and parasitic resistance of the textile or conductive materials, are represented by the resistors $R_{1}$ and $R_{2}$.

The magnetic coupling between the transmitting and the receiving resonators is modeled by the coupling coefficients *k* given by:

(2) $$k=\frac{M}{\sqrt{L_{1}L_{2}}}$$

where *M* is the mutual inductance between the inductors.

IR links have been extensively applied in Wireless Power Transfer (WPT) systems due to their advantages over capacitive coupling and rectenna-based alternatives. These advantages include higher efficiency over short to medium distances and reduced sensitivity to environmental conditions. As a result, inductive resonant coupling is particularly well-suited for applications such as wireless charging of consumer electronics, biomedical implants, and electric vehicles.

However, a major limitation of IR links is their dependence on spatial alignment and distance. Even slight misalignments or changes in distance can lead to a reduction in *k*, resulting in decreased energy transfer performance [40,41].

While such sensitivity is often regarded as a drawback in power transfer applications, it opens up opportunities in sensing and tracking domains. In recent studies, this feature has been successfully exploited to monitor movement by translating variations in the coupling coefficient into positional information. When two resonators are positioned around a joint, such as a knee or elbow, any movement of that joint alters their relative geometry, and thus, the coupling and transmission properties of the link.

This sensing paradigm has several inherent advantages:

- It does not require line-of-sight or visual tracking, making it robust to occlusion or poor lighting conditions;
- The system can operate with minimal electronics since the mechanical motion directly influences the RF response;
- No complex data processing is required, as the relevant information is embedded in the transmission coefficients of the link (i.e., the parameter $S_{21}$ of the scattering matrix of the link).

Such features make IR-based motion tracking attractive for wearable systems, particularly in healthcare, sports performance monitoring, rehabilitation, and ergonomics.

### 2.2. Inductive Resonant Wireless Link in a SIMO Configuration for Posture Monitoring

**Figure 2:**
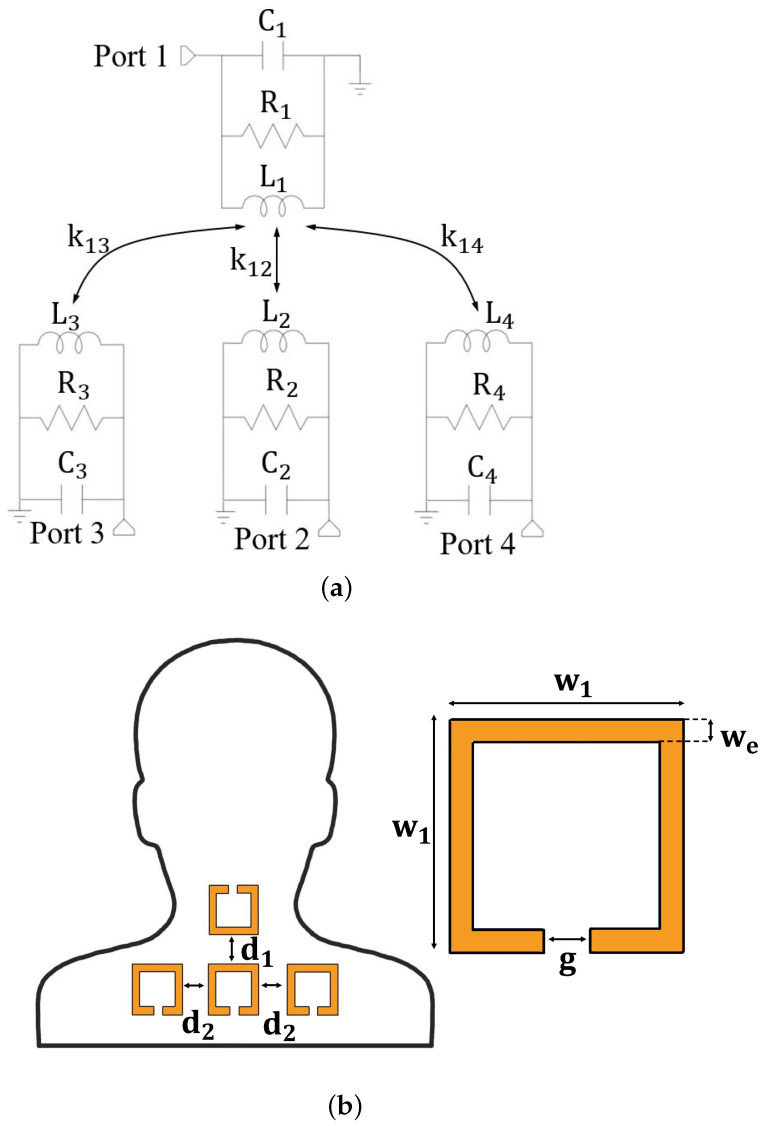
Schematic representation of the proposed IR wireless link for posture monitoring: the link consists of a single transmitter and three receivers. (**a**) Lumped elements equivalent circuit. (**b**) Positioning of the resonators and parameters of the distributed inductors.

The main concept investigated in this work is shown in Figure 2 and consists of using an IR wireless link arranged in a Single Input Multiple Output (SIMO) configuration for posture monitoring, with particular focus on detecting neck misalignments.

In detail, the system includes four magnetically coupled resonators, all tuned to the same resonant frequency. One resonator works as the transmitter and is placed on the back of the neck, while the remaining three act as receivers and are positioned on the left, center, and right regions of the upper back or shoulders.

Each resonator consists of a distributed inductance implemented with a conductive textile and a lumped capacitor in parallel configuration. The resonance frequency $f_{0}$ is given by:

(3) $$f_{0}=\frac{1}{2\pi \sqrt{L_{i}C_{i}}},\;i=\left\{1,2,3,4\right\}$$

The mutual coupling between the transmitter and each receiver is characterized by the coefficient $k_{j1}$:

(4) $$k_{j1}=\frac{M_{j1}}{\sqrt{L_{j}L_{1}}},\;j=\left\{2,3,4\right\}$$

where $M_{j1}$ is the mutual inductance between the transmitter (resonator 1) and the *j*-th receiver.

These coupling coefficients are highly sensitive to posture changes. For instance, when the head tilts forward, backward, or laterally, the spatial configuration of the transmitter with respect to the receivers changes, altering the coupling coefficients and, consequently, the ability of the network to transfer power from the transmitter (resonator 1) to the receivers (resonators 2, 3, 4).

To analyze these variations, the entire system can be modeled as a four-port network and described using the scattering matrix S. Among its elements, the transmission coefficients $S_{j1}$ (j=2,3,4) are of particular interest, as they provide insight into the alignment of the neck relative to the shoulders.

This approach enables non-intrusive posture monitoring. Changes in the transmission coefficients allow for the detection of posture deviations, such as forward head posture or lateral head tilt. Figure 2b illustrates the spatial arrangement of the resonators, optimized for capturing these variations.

Scattering parameters are acquired using a compact and low-power electronic measurement tools. In this work, a NanoVNA was used, a lightweight and battery-operable vector network analyzer. However, for practical and wearable implementations, a custom-designed ultra-low-power board could be developed to miniaturize the system and improve integration into garments. This would allow the realization of an autonomous, fabric-embedded posture monitoring system.

## 3. Materials and Fabrication Techniques

**Figure 3:**
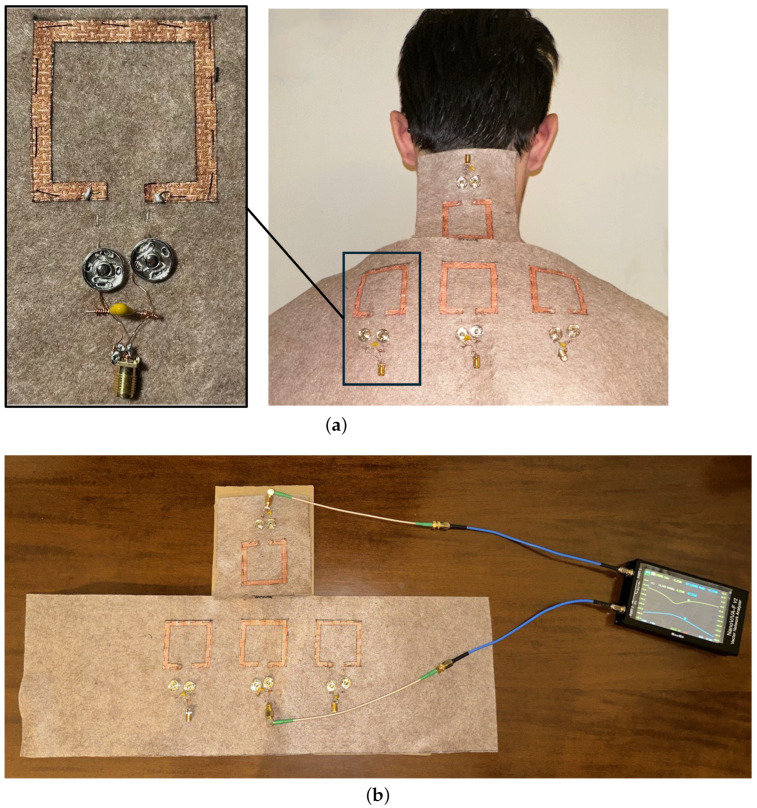
(**a**) Fabricated prototype for posture monitoring. (**b**) Experimental setup adopted for measurements.

The fabricated prototype of the proposed posture monitoring system is illustrated in Figure 3, while the material parameters are provided in Table 1. The prototype consists of four textile-based inductors, each loaded with a lumped capacitor to form resonant circuits. These inductive elements serve as the functional building blocks of the inductive resonant (IR) wireless link operating in a Single Input Multiple Output (SIMO) configuration.

Each inductor was fabricated using a self-adhesive, non-woven conductive textile applied onto a soft and deformable felt fabric substrate. This layered structure ensures both electrical performance and mechanical flexibility, allowing the prototype to conform comfortably to the shape of the human body. The conductive fabric used is Kiel-SK-96 by Shieldex [42], a thermally bonded non-woven structure coated with pure copper. Thanks to its high copper content (approximately 50%), this fabric exhibits excellent electrical conductivity, with a surface resistance of less than 0.02 Ω/square. This makes it especially suitable for realizing low-loss inductive structures compatible with wearable electronics.

Electrical connections between the textile inductors and the measurement circuitry were implemented using a combination of metal snap buttons and conductive yarn. The snap buttons provide a key advantage by allowing non-washable electronic components to be easily detached from the textile structure.

The conductive yarn used is the 235/36 x4 HCB by Shieldex [43], characterized by a round cross-section and a nominal resistivity of 40 Ω/m ± 10 Ω/m. This ensures stable electrical performance while preserving the flexibility typical of textile materials.

Although specific washing tests were not conducted on the current prototype, the design prioritizes modularity and washability by physically separating the passive textile resonators from the active electronic measurement board. This allows the textile component to be safely washed without risking damage to the electronics.

Regarding the durability of the textile components, the washability and resilience of similar conductive non-woven fabrics were extensively analyzed in a previous work [44]. In particular, in [44], it was demonstrated that these materials retain their electrical performance and structural integrity even after multiple washing and ironing cycles, confirming their suitability for washable wearable applications.

For signal interfacing and characterization, lumped capacitors and SubMiniature version A (SMA) coaxial connectors were used to connect the textile inductors to a portable Vector Network Analyzer (VNA). Specifically, a NanoVNA-F V2 device by SISJOINT was adopted for measuring the scattering parameters of the system. However, in a future wearable implementation, these bulky connectors can be eliminated by designing a compact and dedicated electronic board. This custom board could include both the lumped capacitors and the RF measurement circuitry, potentially integrated with a low-power wireless module (e.g., Bluetooth Low Energy) for continuous or periodic data transmission to a mobile device or cloud server.

The design process began with the fabrication of a single textile-based inductive ring, whose geometry was defined to satisfy placement constraints. Specifically, the inductor had to be small and flexible enough to conform to the human body, particularly to fit one resonator on the neck and three others on the shoulders, as illustrated in Figure 2. Among the design parameters, the resonator size (i.e., the parameter $w_{1}$ illustrated in Figure 2) was especially critical, as it had to ensure sufficient coupling area while maintaining mechanical comfort and flexibility. A complete list of the geometrical parameters adopted in the final prototype is reported in Table 2, with reference to the schematic in Figure 2.

Once the inductor geometry was fixed, the optimization process continued with tuning the resonance frequency. To achieve resonance around 13.56 MHz—within the ISM band and suitable for low-power wireless systems—a set of measurements was performed by connecting different lumped capacitor values to the textile inductor. The reflection coefficient $S_{11}$ was measured using a portable NanoVNA to verify the resonance frequency for each capacitor value. This empirical tuning procedure led to the selection of a 1 nF capacitor, which resulted in a resonance frequency of approximately 13.12 MHz. According to this result and referring to the equivalent circuit illustrated in Figure 2, the values of the equivalent lumped parameters for the textile inductors are L=147 nH, R=33.56 Ω.

**Figure 4:**
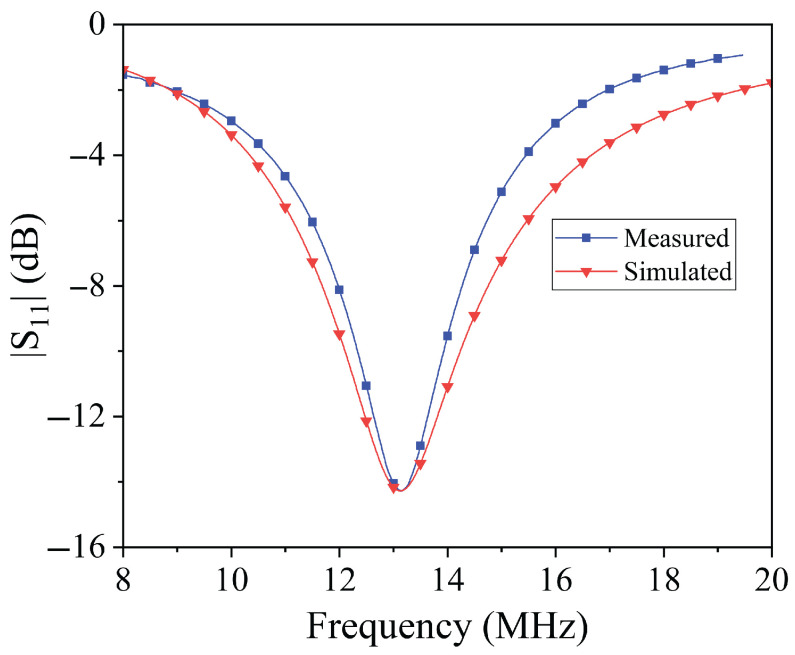
Reflection coefficient of the single resonator: comparison between simulated data obtained for the equivalent circuit and measured data.

The reflection coefficient ($S_{11}$) of the single resonator, shown in Figure 4, demonstrates good agreement between the experimental data and the simulation results obtained using a circuital simulator (AWR Design Environment by Cadence [45]). This agreement confirms the estimated values of the equivalent circuit parameters. While the match is not perfect—due to the simplified nature of the model, which neglects parasitic effects introduced by connectors and layout—it provides a reliable basis for further design adjustments. For example, knowing the inductance value allows straightforward reconfiguration of the resonator’s operating frequency by selecting the appropriate capacitance.

After the successful characterization of the single element, the complete four-resonator system was assembled (see Figure 3). The prototype was then subjected to experimental testing to evaluate its ability to detect changes in posture. Various head and neck positions were reproduced and the resulting transmission coefficients were measured using the VNA. To guide the subject in reproducing specific head tilt angles (both forward and lateral), a cardboard reference structure with angular markings was employed. This setup allowed the subject to align their head to predefined angles (e.g., 0°, 15°, 30°, 37°, 45°) during the measurements, ensuring consistency across trials. The achieved results, discussed in detail in the following section, demonstrate the effectiveness of the proposed inductive link configuration for posture recognition based on S-parameter measurements.

The proposed fabrication approach emphasizes modularity, washability, and mechanical comfort, all of which are essential for real-world deployment in wearable applications. Future iterations of the prototype could explore further miniaturization of the electronics, flexible encapsulation of the measurement board, and integration into garments or accessories such as collars, shoulder pads, or posture-corrective wearables.

## 4. Experimental Results

To validate the operating principle of the proposed posture monitoring system, comprehensive measurements of the transmission coefficients were conducted for various neck tilt angles, including both forward and lateral displacements. For each posture configuration, the complete set of transmission parameters $S_{j1}$ with j=2,3,4 was acquired, corresponding to the signal transmitted from the resonator on the neck (port 1) to each of the three shoulder-mounted resonators (ports 2, 3, and 4).

The measurements were performed using the *NanoVNA-F V2* by SISJOINT, a two-port VNA capable of measuring scattering parameters of two-port electrical networks. As such, for each posture, three sequential measurements were carried out by connecting port 1 of the VNA to the neck resonator (resonator 1) and sequentially connecting port 2 to each of the shoulder resonators (resonators 2, 3, and 4). This procedure was repeated for each tilt angle to obtain the complete transmission profile associated with different postural configurations.

### 4.1. Forward Tilt Analysis

When the neck undergoes a forward tilt, the primary observable effect is a variation in the amplitude of the transmission coefficient $S_{21}$. This behavior is expected based on the equivalent circuit model depicted in Figure 2, where a forward displacement predominantly alters the coupling coefficient $k_{21}$ between resonator 1 (placed on the neck) and resonator 2 (positioned on the shoulder). As the neck bends forward, the distance and alignment between these two resonators change, resulting in a measurable change in mutual inductance, and thus, in the transmission coefficient amplitude.

Conversely, the coupling coefficients between resonator 1 and the other two resonators (3 and 4, located symmetrically on the shoulders)—i.e., $k_{31}$ and $k_{41}$—are expected to exhibit a symmetric and comparatively minor variation during forward tilt. This is consistent with the symmetric physical arrangement of the resonators and the relative stability of their geometric relationships during such movement.

**Figure 5:**
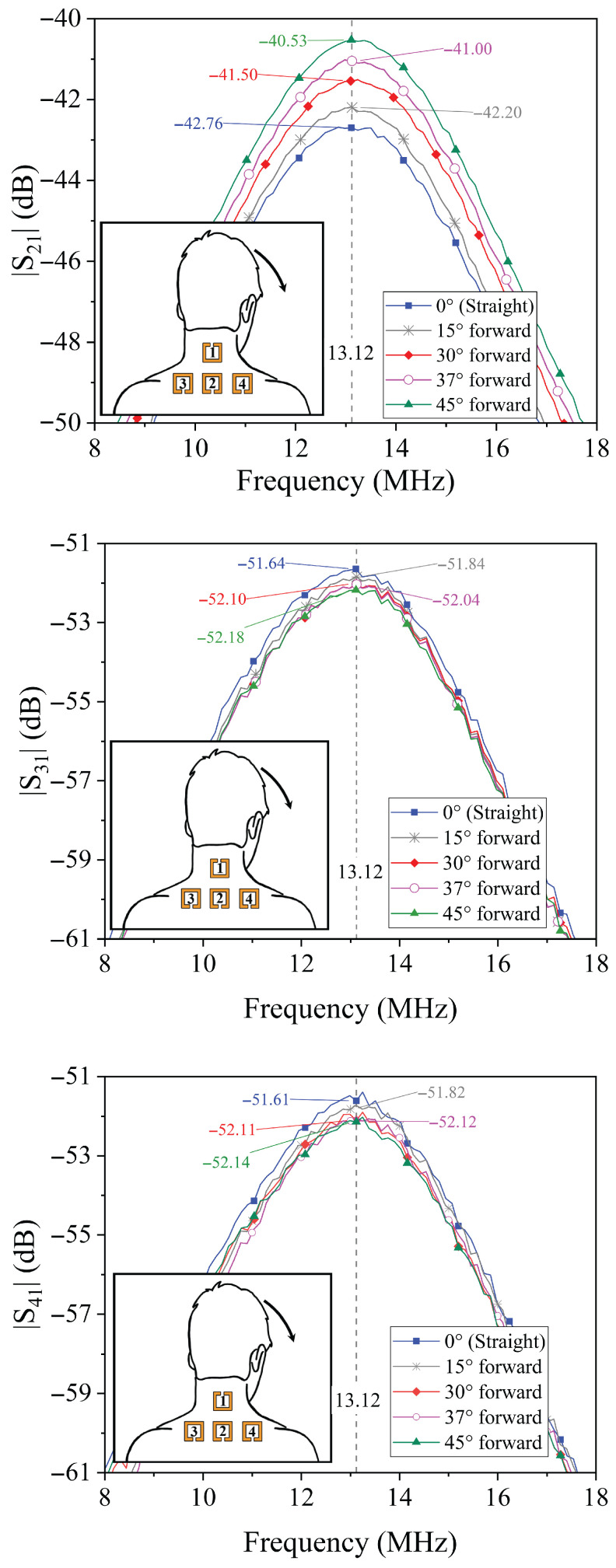
Results obtained for the prototype illustrated in Figure 3. Measured data of the transmission coefficients for different values of the forward tilt angle.

Figure 5 shows the measured transmission coefficients for neck forward tilts of 15°, 30°, 37°, and 45°. It is evident that the amplitude of $S_{21}$ at the resonance frequency increases monotonically with the forward tilt angle, indicating a direct correlation between neck position and $S_{21}$ amplitude. This trend confirms the system’s sensitivity to forward neck movements. Similarly, the amplitudes of $S_{31}$ and $S_{41}$ exhibit a slight decrease when compared to the neutral neck posture, reflecting the expected symmetric behavior and further supporting the model.

### 4.2. Lateral Tilt Analysis

In the case of lateral tilts, the transmission coefficients $S_{31}$ and $S_{41}$ serve as the key indicators. A lateral tilt to the left causes the coupling coefficient between resonator 1 and resonator 3 ($k_{31}$) to increase due to their closer proximity or improved alignment, while simultaneously causing a decrease in the coupling coefficient $k_{41}$ between resonator 1 and resonator 4. The opposite behavior is observed for a lateral tilt to the right, where $k_{41}$ increases and $k_{31}$ decreases.

**Figure 6:**
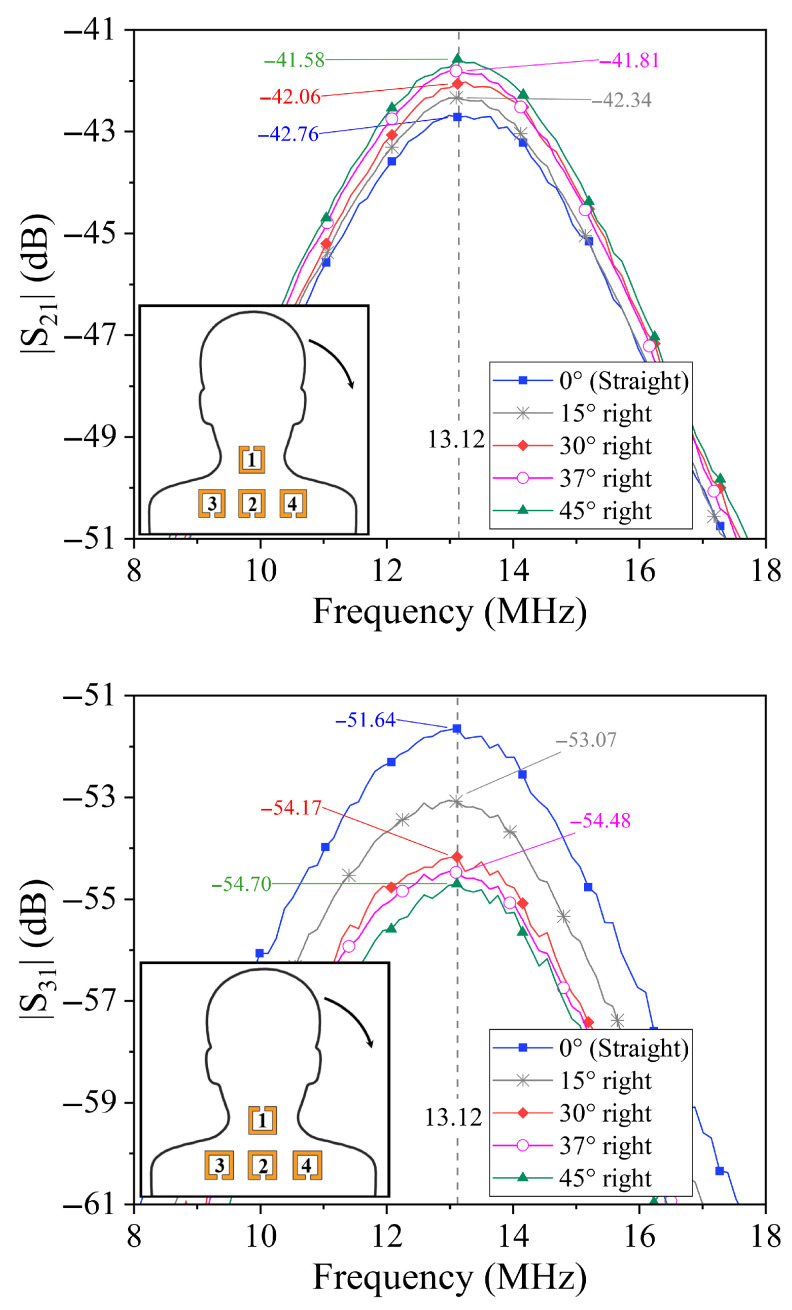

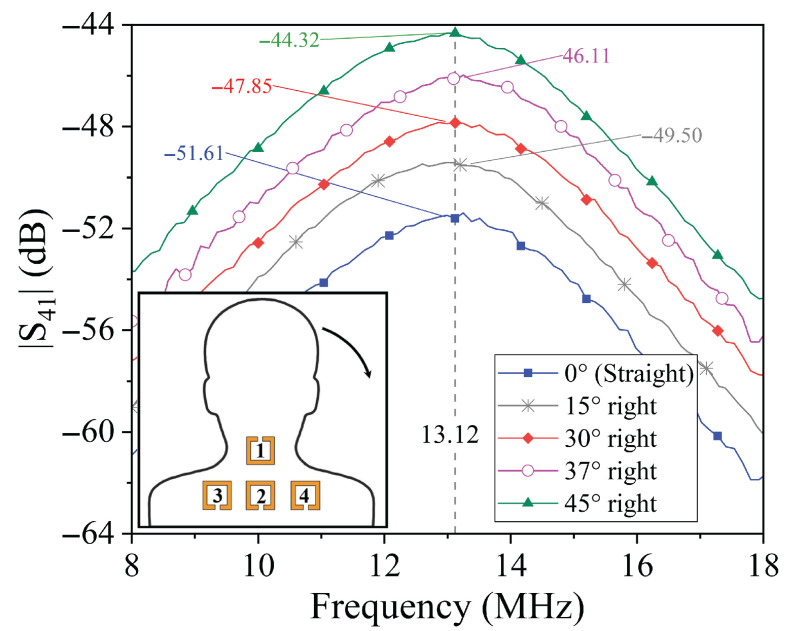
Results obtained for the prototype illustrated in Figure 3. Measured data of the transmission coefficients for different values of the neck lateral bending angle to the right.

**Figure 7:**
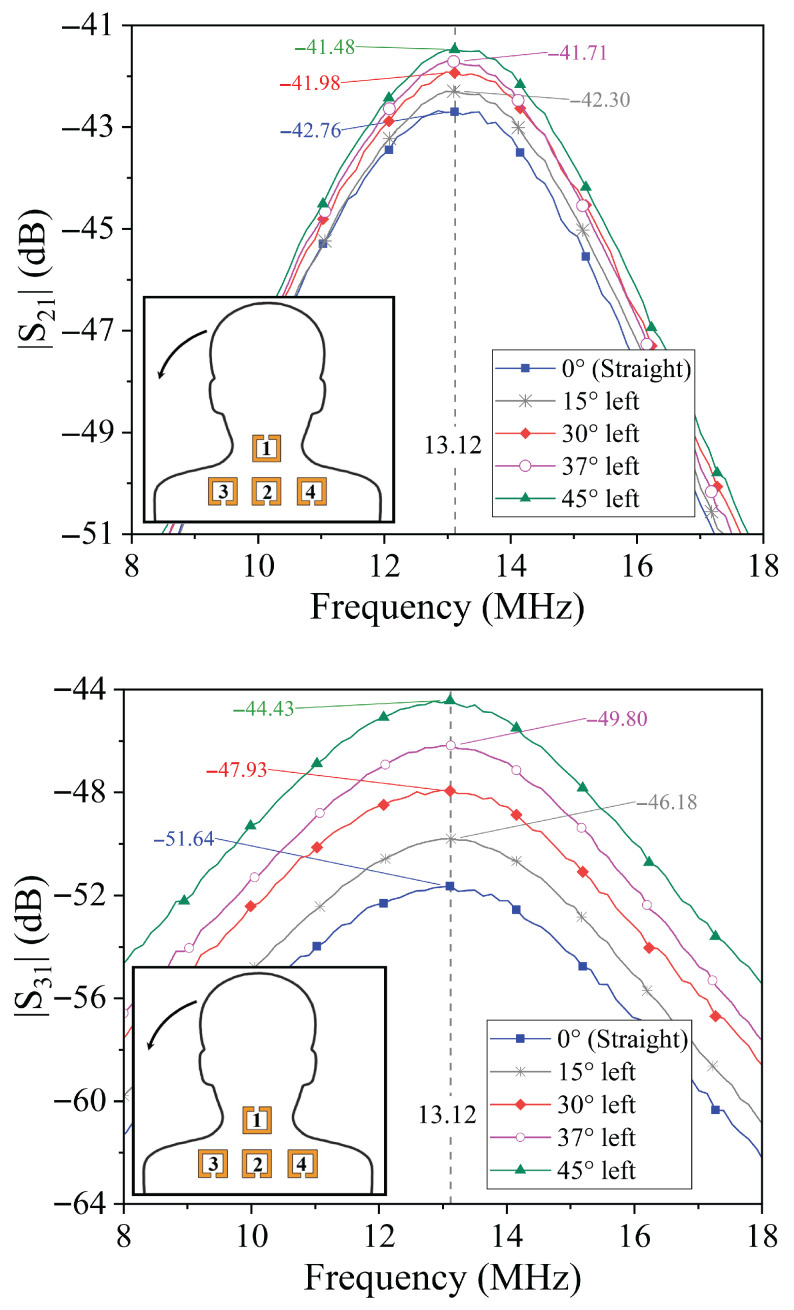

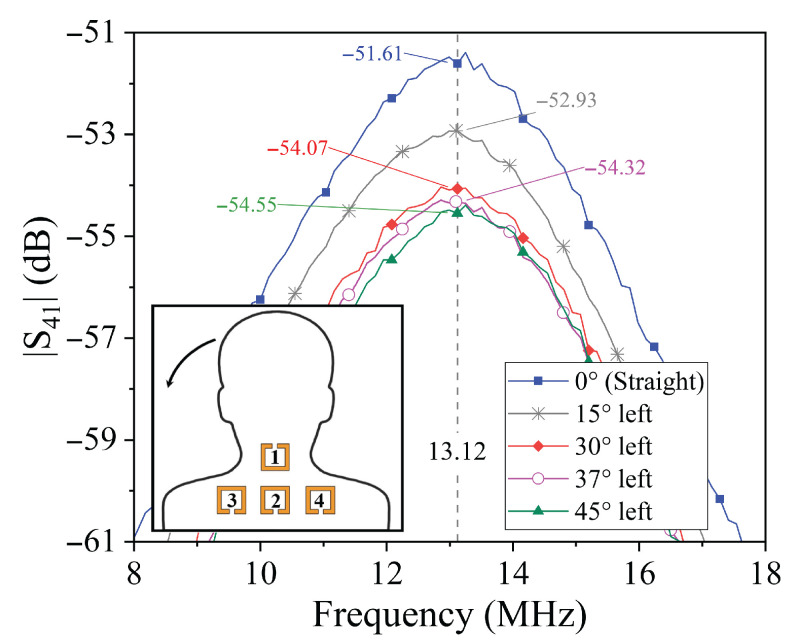
Results obtained for the prototype illustrated in Figure 3. Measured data of the transmission coefficients for different values of the neck lateral bending angle to the left.

The transmission coefficient amplitudes measured under lateral tilts to both left and right are reported in Figure 6 and Figure 7. As expected, $S_{31}$ and $S_{41}$ exhibit opposite trends: as the neck tilts to the right, $S_{31}$ decreases while $S_{41}$ increases; for left tilts, these behaviors are reversed. This anti-symmetric response facilitates unambiguous detection of the direction of lateral tilt.

Interestingly, the amplitude of $S_{21}$ shows a slight increase with increasing lateral tilt angle regardless of the direction (left or right), as shown in the figures. However, this parameter alone is insufficient to distinguish lateral tilt direction, highlighting the importance of combined analysis of $S_{31}$ and $S_{41}$ for robust posture detection.

### 4.3. Discussion

The results obtained for the measured transmission coefficients clearly demonstrate the possibility of correlating these values with the position of the neck.

**Figure 8:**
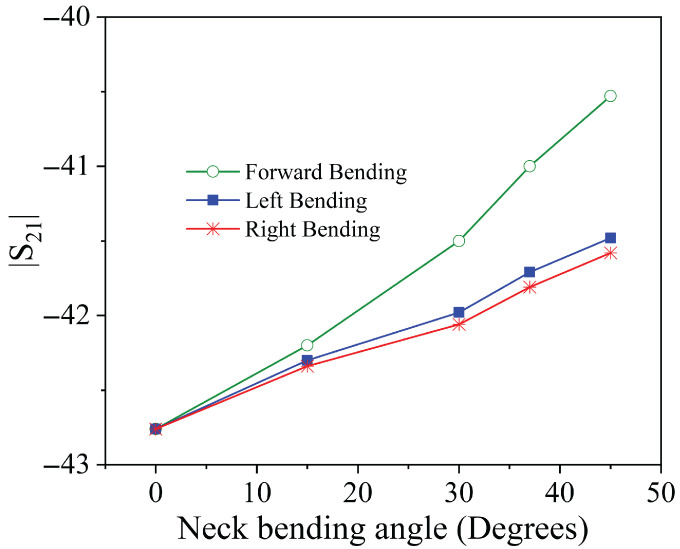
Amplitude of the transmission coefficient $S_{21}$ at the frequency of resonance as a function of the neck bending angle.

**Figure 9:**
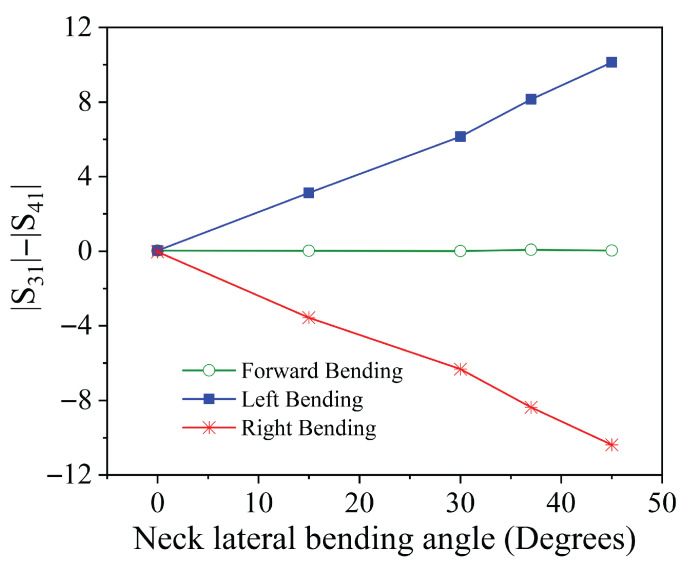
Difference of the amplitudes of the transmission coefficients $S_{31}$ and $S_{41}$ at the frequency of resonance as a function of the neck bending angle.

To illustrate this potential, Figure 8 presents the magnitude of the $S_{21}$ parameter at the resonance frequency as a function of the bending angle for both forward and lateral neck movements. Similarly, Figure 9 displays the difference between the amplitudes of the parameters $S_{31}$ and $S_{41}$, also evaluated at the resonance frequency, relative to the same angular variations.

Analysis of these figures reveals that the neck posture can be reliably inferred by combining the information from $S_{21}$ and the difference between $S_{31}$ and $S_{41}$. Specifically, the amplitude of $S_{21}$ increases in response to both forward and lateral tilts. However, the increase is more pronounced in the case of forward tilt. On its own, this parameter does not allow discrimination between the two types of motion.

The key to disambiguating the movement lies in the behavior of $S_{31}$ and $S_{41}$. In forward tilts, these two parameters exhibit a similar decrease in amplitude, resulting in a negligible difference. In contrast, during lateral tilts, their responses diverge significantly, with the relative amplitude difference indicating both the presence and the direction of the movement (left or right).

Therefore, by jointly analyzing the magnitude of $S_{21}$ and the differential behavior of $S_{31}$ and $S_{41}$, it becomes possible to clearly distinguish between forward and lateral neck tilts. This combined approach enables accurate and low-complexity classification of neck posture, while also providing a quantitative indication of the direction and extent of the movement.

### 4.4. Post-Processing and Practical Implementation Considerations

The results presented in this paper were obtained using a laboratory prototype and a commercial portable Vector Network Analyzer (a NanoVNA), which directly provides the scattering parameters $S_{j1}$ without further post-processing. These parameters are inherently linked to the relative positions of the resonators and, as demonstrated, can be effectively used to infer the user’s neck posture. In a future wearable implementation, a dedicated and miniaturized measurement board—essentially a simplified NanoVNA without a display—could be embedded into the garment. This compact system would acquire the $S_{j1}$ coefficients and either process them locally using an embedded microcontroller or transmit them wirelessly (e.g., via Bluetooth) to a mobile device.

A possible operational workflow would include an initial calibration phase, where the system records transmission coefficients corresponding to known reference postures (e.g., upright, 30° forward tilt, 45° lateral tilt, etc.). These reference values can be used to define posture categories and thresholds. During regular use, the system would continuously compare real-time $S_{j1}$ values against the calibrated reference set to classify the current posture. A simple algorithm could then monitor the duration for which an incorrect or suboptimal posture is maintained. If this condition persists beyond a predefined threshold (e.g., several minutes), the system could generate a corrective alert, such as a vibration, sound, or notification on a paired mobile device.

Alternatively, the system could offer on-demand feedback to the user, for example via a mobile application that displays current posture status or logs daily posture habits. This flexibility allows the device to function both as a real-time correction tool and as a posture awareness aid, supporting long-term ergonomic health and user training.

## 5. Conclusions and Future Work

In this paper, a proof-of-concept system based on a wireless inductive resonant (IR) link in a SIMO configuration was presented for wearable posture monitoring. The proposed approach exploits variations in mutual coupling between textile-integrated resonators to detect changes in neck posture, including both forward and lateral tilts.

The results demonstrate that posture-related information can be effectively extracted from direct measurements of the scattering parameters, without requiring complex post-processing or high-complexity signal fusion algorithms. This simplicity translates into reduced system complexity, lower power consumption, and improved robustness against measurement errors, especially when compared to conventional systems based on inertial sensors.

The hardware configuration—comprising only passive textile resonators and a compact, detachable electronic board—makes the system highly suitable for integration into wearable garments, ensuring comfort, modularity, and ease of maintenance. Additionally, its independence from externally equipped environments enhances portability and usability in real-world scenarios.

Regarding external influences, the system is expected to be inherently robust to electromagnetic interference and to the proximity of metallic objects, due to the near-field nature of the inductive coupling. Nevertheless, to prevent possible malfunctions caused by direct contact with conductive materials, the textile resonators could be covered with a thin insulating fabric layer. As for electromagnetic interference from external sources, this aspect will require further investigation. Should any performance degradation be observed in realistic environments, mitigation strategies—such as shielding or filtering techniques—could be implemented to ensure measurement reliability.

From the perspective of energy consumption, the system benefits from the inherently low power requirements of S-parameter measurement. While the current prototype uses a commercial nanoVNA, future implementations will involve a custom, miniaturized board optimized for power efficiency. Based on estimates derived from commercial devices (e.g., NanoVNA), expected power consumption could remain below 200–300 mW, allowing for several hours of operation with small wearable batteries.

Future developments will focus on the miniaturization and optimization of the electronic measurement unit, the implementation of a dedicated posture classification algorithm (executed either on-board or via a mobile device), and the validation of the system in real-world scenarios. These efforts will support the assessment of performance metrics and enable comparative studies with existing wearable technologies.

Overall, the presented results confirm the potential of inductive coupling technologies to enable the next generation of wearable, low-power, and user-friendly systems for continuous posture monitoring.

## Figures and Tables

**Table 1:** Materials adopted for the fabrication of the inductors.

Material	Electrical Properties (Resistivity)
Conductive yarn	40 Ω/m ± 10 Ω/m
Conductive non-woven fabric	<0.02 Ω/square

**Table 2:** Dimensions of the inductors (see Figure 2 for the meaning of the parameters).

$w_{1}$ (cm)	$w_{e}$ (cm)	g (cm)	$d_{1}$ (cm)	$d_{2}$ (cm)
5	0.5	1	4	3

## Data Availability

The original contributions presented in this study are included in the article. Further inquiries can be directed to the corresponding author.
